# Probiotics by Modulating Gut–Brain Axis Together With Brivaracetam Mitigate Seizure Progression, Behavioral Incongruities, and Prevented Neurodegeneration in Pentylenetetrazole‐Kindled Mice

**DOI:** 10.1111/cns.70078

**Published:** 2024-10-29

**Authors:** Muhammad Usman Shakoor, Fashwa Khan Tareen, Zohabia Rehman, Khaled Ahmed Saghir, Waseem Ashraf, Syed Muhammad Muneeb Anjum, Tanveer Ahmad, Faleh Alqahtani, Imran Imran

**Affiliations:** ^1^ Department of Pharmacology, Faculty of Pharmacy Bahauddin Zakariya University Multan Pakistan; ^2^ The Institute of Pharmaceutical Sciences University of Veterinary & Animal Sciences Lahore Pakistan; ^3^ Institut Pour l'Avancée Des Biosciences, Centre de Recherche UGA/INSERM U1209/CNRS 5309 Université Grenoble Alpes Grenoble France; ^4^ Department of Pharmacology and Toxicology, College of Pharmacy King Saud University Riyadh Saudi Arabia

**Keywords:** EEG, epilepsy, gut–brain axis, Nissl's staining, oxidative stress, probiotics, PTZ kindling

## Abstract

**Background:**

The microbiota–gut–brain axis (MGBA) is a central nexus that integrates higher cognitive and emotional centers of the central nervous system (CNS) within the intricate functioning of the intestine. Accumulating evidence suggests that dysbiosis in the taxonomic diversity of gut flora plays a salient role in the progression of epilepsy and comorbid secondary complications.

**Methods:**

In the current study, we investigated the impact of long‐term oral bacteriotherapy (probiotics; 10 mL/kg; 10^9^ colony‐forming unit/ml) as an adjunctive treatment intervention with brivaracetam (BRV; 10 mg/kg) over 21 days on pentylenetetrazole (PTZ) induced augmented epileptic response and associated electrographical and behavioral perturbations in mice. Moreover, we also unveiled antioxidant capacity and histopathologic changes in treated versus non‐treated animals.

**Results:**

Results revealed combination increases seizure threshold and prevented high ictal spiking. Additionally, it alleviated PTZ‐induced neuropsychiatric disturbances such as anxiety and depressive‐like phenotype along with cognitive deficits. Furthermore, dual therapy prompted physiological oxidant/antioxidant balance as evidenced by increased activity of antioxidant enzymes (SOD and catalase) and reduced levels of oxidative stressor (MDA). This therapeutic intervention with commensal species suppressed network‐driven neuroinflammation and preserved normal cytoarchitecture with intact morphology in the pyramidal layers of cornu ammonis (CA1 and CA3).

**Conclusion:**

Our study provides supporting evidence for the use of probiotics as adjunctive therapy with anti‐seizure medications to modulate epileptogenic processes and related multimorbidities, particularly in individuals with drug‐resistant seizures.

## Graphical abstract

Outcomes from this study provide supportive evidence in favor of probiotic‐rich supplementation as an adjunctive therapy with existing antiseizure medications as these commensal bacteria might provide prophylaxis against intractable seizures, particularly in predisposed individuals.

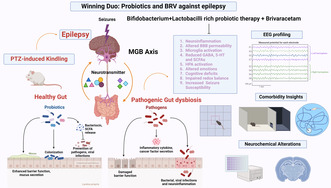

## 1 Introduction

Venerated as a second genome in the human ecosystem, the gut microbiota is implicated in a constellation of pathophysiological intricate processes convoluting those related to brain function and behavior [1]. Microbes have invariably been a pivotal part of human life and the human gut harbors diverse commensal microbial community particularly anaerobic bacteria that captivate this environs; besides protozoa, fungi, archaea, and viruses which primarily aid in the regulation of equivocal microecological parity of metabolism [2]. A recent tandem of evidence supports the co‐evolution between the human host and microbial species (human biome) establishing beneficial mutualistic symbiosis. Thus, owing to its extensive genetic and metabolic capacity, the microbe connectome predicates its potential ubiquity in practically every critical aspect of human biology encompassing overall health maintenance, growth, aging, and prognosis of disease.

A growing consensus of literature continues to illustrate the biological eminence of gut microbiota and unveils bidirectional interplay between the central nervous system and microbiome forming the microbiota–gut–brain axis (MGBA). Through the MGBA communication network, commensal gut species mediates complex physiological processes critical for brain health and mood via modulation of immune pathways, vagus nerve, neuroendocrine system, and circulatory system by synthesis of neuroactive metabolites such as short‐chain fatty acids (SCFAs), tryptophan metabolites, bile acid metabolites, neuropeptides, and hormones [3]. Drifts in the taxonomic distribution of gut microbiota have been substantially linked with the development of impaired social behavior, memory deficits, anxiety, mood disorders, reduced psychomotor activity as well as increased susceptibility toward neurodegenerative disorders as the brain profoundly depends on intestinal microbes for vital metabolic products. Furthermore, intestinal flora is implicated in the stimulation of neurotransmitters such as GABA, serotonin, and dopamine and amino acids (tyrosine and tryptophan). Neurotransmitters cannot cross the BBB and usually act locally via the enteric nervous system and thus have an indirect influence on the brain. In contrast amino acids can traverse across BBB after entering into systemic circulation and are harnessed as neurotransmitter precursors which can directly affect CNS homeostasis [4]. Therefore, the preclinical and clinical studies have corroborated compelling mechanistic insights highlighting the increased drive in neurodegenerative disorders pathophysiogenesis (i.e., autism, Parkinson's, Alzheimer's disease, and epilepsy) and metabolic dysbiosis [5].

Epilepsy is a circuit disorder accompanied by hypersychronized high‐frequency recurrent neuronal bursts but also by exorbitant psychosocial consequences, and comorbid detrimental somatic and behavioral perturbations [6]. The pathogenic neurobiological modifications entailing epileptogenesis are still enormously cryptic but assumed to append altered ion transport in the neuronal membrane, imbalance between excitatory and inhibitory synaptic inputs impacting neuronal homeostasis, neuronal circuit's reorganization, neurodegeneration, axonal sprouting, and impaired oxidant/antioxidant factors [7]. Furthermore, pharmacoresistance to contemporary anti‐seizure medications (ASMs), the augmented prevalence of active epilepsy globally, and the heterogeneous semiology of ictal episodes along with the genesis of coexisting multimorbidities particularly cognitive dysfunction suggest more intensive epilepsy research to devise plausible therapeutic interventions. Chemical kindling triggered via repetitive administration of the sub‐threshold dose of pentylenetetrazole (PTZ), a noncompetitive GABA_A_ receptor antagonist, is a central and handy murine model of epilepsy widely used to hunt seizure‐provoked neuronal synchrony, anatomical changes, regulatory modulator system, oxidative stress, interneuron loss, and associated progression of systemic alterations [8].

Metabolic dysbiosis imparts a basis for the upregulation of systemic immune pathways which can ignite mirrored injury in the CNS while neurological impairments in lieu may elicit systemic inflammation leading toward fluctuations in microecological gut balance. This set of repercussions can supplement the foundation for hyperexcitability, progression of epileptogenesis, and associated multimorbidities supporting the intermediary role of adaptive and innate and autoimmune responses in the MGBA in epilepsy as microbes can identify pro and preinflammatory cytokines and chemokines via pattern recognition receptors (PRR) [9]. Moreover, blood–brain barrier disruption further generates a self‐sustaining cycle in which seizure and microbial dysbiosis influence each other via instigating inflammatory cascades in the context of epilepsy [10]. Another key element of MGBA is the hypothalamic–pituitary–adrenal axis (HPA) as well as the endocannabinoid system whose relative collusion in stress‐instigated response and neuromodulation may impact CNS physiological functions. Nonetheless, MGBA is a multi‐pathway prompted network, still in need of full‐scale comprehension. Innovative therapeutic strategies chiefly aiming to dominate/control these complex interconnected pathways may lead toward robust management of intractable seizures.

Probiotics are typified by the World Health Organization (WHO) as diverse viable microorganisms that when given in sufficient therapeutic doses accord symbiotic health benefits to the host prompting a favorable microbial state in the gut [11]. This can significantly influence the disease progression in patients with refractory epilepsy as probiotics can increase convulsive threshold via regulation of GABA and glutamate levels in the host brain, reduction in proinflammatory cytokines, and restoration of physiological oxidant/antioxidant levels further supporting the protective role of microbiota in epileptogenic process. Brivaracetam (BRV) on the other hand is the new third‐generation broad‐spectrum anti‐seizure medication with a selective affinity for synaptic vesicle glycoprotein 2A (SV2A) [12].

Considering the high incidence rate of drug‐resistant epilepsy, further research on adjunctive pharmacological regimens may provide the useful in‐depth mapping of network rewiring and etiopathogenesis in nascent epileptogenic foci. The current study aims to unravel the protective impact of MGBA modulation by chronic probiotic supplementation as adjunctive therapy with BRV on PTZ‐induced augmented epileptic response, interneuron loss, redox stress, and the development of related neuropsychiatric disturbances.

## 2 Materials and Methods

### 2.1 Animals

Male BALB/c mice weighing 18–25 g were housed in cages made up of polycarbonate in the Faculty of Pharmacy, Department of Pharmacology, Bahauddin Zakariya University Multan, Pakistan. The animals were provided with standard rodent food and water under well‐controlled hygienic environment maintained at 23 ± 2°C. These mice were given the 12 h light/dark cycle and all experiments were carried out during the daytime (9:00 a.m.–5:00 p.m.). G*Power 3.1.9.4 software was utilized for sample size calculation. We determined that 10 animals per allocated treatment were required for electrographical, behavioral, neurochemical, and histopathological characterization given the effect size 1.795, the alpha error was 0.05%, and power (1−β err prob) was 0.95 and the calculation assumed two‐tailed test. Mice were randomly allocated to the treatment groups by a third person with no significant difference in age and body weight in the blocks of 10 for six groups as elucidated in animal grouping and dosing protocol below to minimize bias. Ethical approval was obtained from the Departmental Ethical Committee for the utilization of laboratory animals (EC/06‐PHL‐S‐23; dated October 17, 2023) of the Department of Pharmacology, B.Z. University, Multan for these in vivo experiments. All experiments were commenced as per ARRIVE guidelines.

### 2.2 Drugs and Chemicals

Brivaracetam (BRV, Lot. no. NP17‐13‐2111003) was obtained from Zhejiang Eazy Pharmchem and PTZ from Sigma Aldrich, while diazepam (Dia) was procured from Rosche Pharma Pakistan as a commercially available ready‐to‐use injectable formulation (Valium 10 mg/2 mL). PTZ (40 mg/kg), BRV (10 mg/kg), and Dia (1 mg/kg) were dispensed in normal saline. Bacteriotherapy (Ecotec) containing *Lactobacillus* sp., *Bifidobacterium* sp., and *streptococcus* sp. having a 10^9^ colony forming unit (CFU), and one sachet was dissolved in 10 mL of normal saline and was orally given at the volume of 10 mL/kg. Information regarding drug solubility and dose was obtained from the previously published literature [13, 14].

### 2.3 Stereotaxic Surgery for Cortical Electrode Implantation

Stereotaxic surgery for cortical electrode implantation was performed in anesthetized animals with the help of intraperitoneal injection of ketamine (87.5 mg/kg) and xylazine (12.5 mg/kg) cocktail. The animal's consciousness was checked by toe pinching withdrawal reflex and under complete anesthesia, its head was fixed in a stereotaxic apparatus (Stoelting, USA) that was provided with a heating pad for thermoregulation during the surgical procedure. After shaving and cleaning the head with 70% ethanol, a 2 cm long incision was made to expose the skull. Soft tissue/periosteum of the skull was cleaned and then four small burr holes (1 mm) were gently made gently an electrical driller to avoid deep drilling. Custom‐made cortical tripolar Arduino electrodes (2.54 mm) round right‐angle female pin header used as a connector and designed to match 0.1′ (2.54 mm) male headers and custom‐made EEG cables were used to acquire EEG signals from the positive, negative and ground terminals. The implantation of tripolar cortical electrodes was made at AP + 3 mm; LL ± 1.5 mm from bregma, and taking one screw as a reference electrode implanted at AP—2 mm; L—1.5 mm [15]. Implanted electrodes were secured by applying dental cement and 0.9% normal saline bolus was given to each animal. All the animals were observed for their normal movement and behavior after surgery and a period of 1 week was provided for post‐operative recovery before the experiment.

### 2.4 Animal Grouping and Dosing Protocol

A total of 60 BALB/c mice were randomly chosen and sorted into six groups (*n* = 36 without electrodes for behavioral tests and neurochemical assays and *n* = 24 with cortical electrode implants for EEG).

The 36 animals (*n* = 6) without electrode implants and 24 animals with EEG electrode implants (*n* = 4) were grouped as follows:

- Group 1: healthy control (normal saline 1 mL/kg, i.p. daily),
- Group 2: PTZ 40; negative control (PTZ 40 mg/kg, i.p. on every other day; 11 injections),
- Group 3: DIA 1; positive control (diazepam 1 mg/kg, i.p. daily + PTZ),
- Group 4: PRO 10 (probiotics 10 mL/kg, i.g. daily + PTZ),
- Group 5: BRV 10 (brivaracetam 10 mg/kg, i.p. daily + PTZ), and
- Group 6: BRV 10 + PRO 10 (BRV 10 mg/kg, i.p. + probiotic 10 mL/kg daily + PTZ). All groups were given their respective treatment daily for 21 days.

### 2.5 PTZ‐Kindling Protocol

All BALB/c mice were administered PTZ intraperitoneally in subconvulsive doses of 40 mg/kg on alternate days (11 injections) except the healthy control group. All animals were observed behaviorally for at least 30 min after PTZ injection for seizure score. Kindling and intensity of seizures were assessed by a modified Racine scale.

- Stage 0: No response.
- Stage 1: Behavioral arrest, ipsilateral eye blinking.
- Stage 2: Head nodding.
- Stage 3: Forelimb clonus.
- Stage 4: Rearing.
- Stage 5: Rearing and falling.
- Stage 6: Death.

**FIGURE 1:**
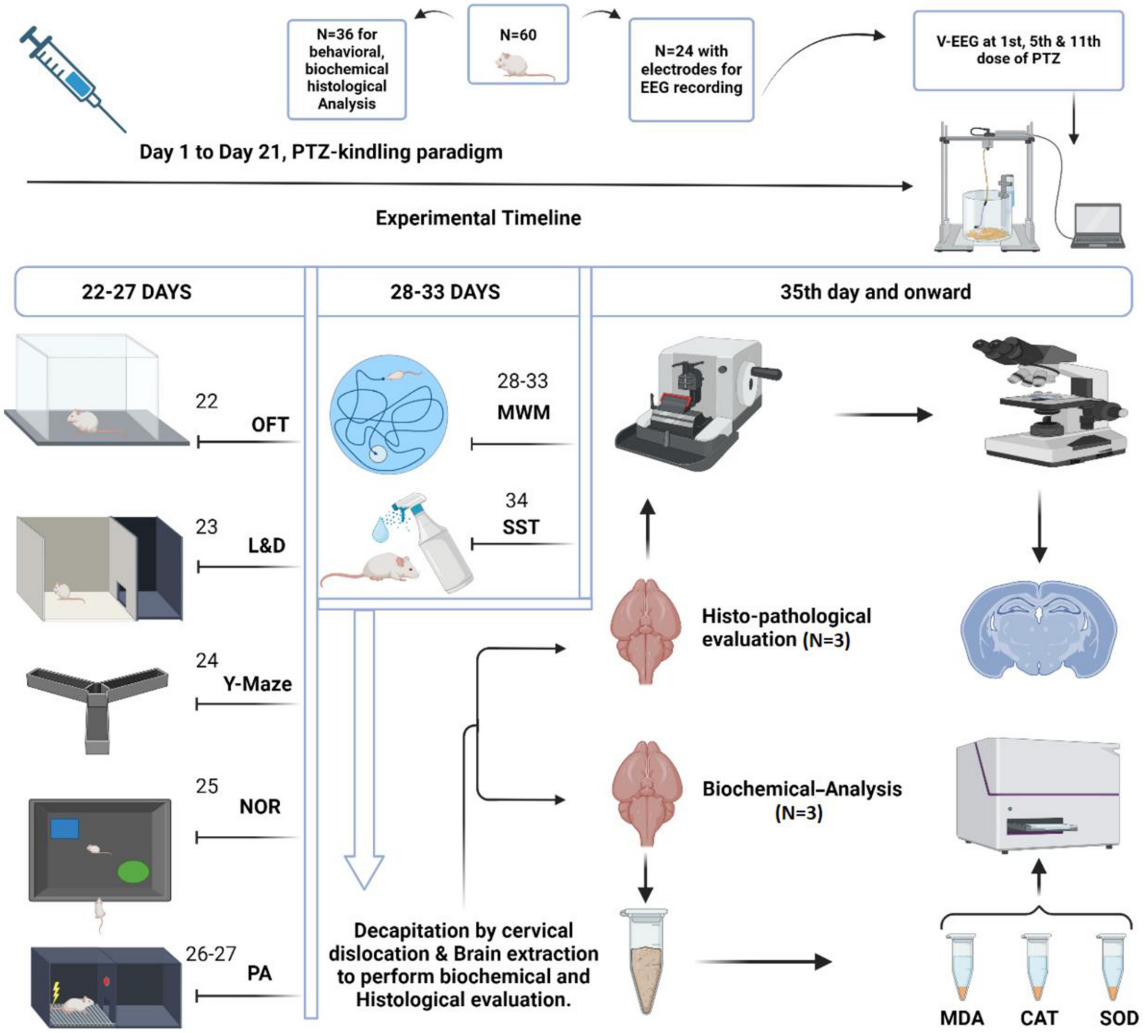
Experimental timeline for PTZ kindling and associated secondary comorbidities to assess the impact of BRV 10 + PRO 1 dual therapy on epileptogenic processes and plausible improvement in neuronal survival.

Animals that showed four or five‐stage seizures consecutively by the 9th–11th PTZ injections were considered as fully kindled [16]. After 21 days of dosing, animals were tested for neurobehavioral deficits in the isolated noiseless facility in the time window 9:00 a.m.–5:00 p.m. in a series of least to most stressful tests after 30 min–1 h of acclimatization. All the neurobehavioral tests were commenced by the experimenter completely blinded toward the current treatment protocol to ensure data coherence and transparency. Immediately, after the end of the neurobehavioral assessment, all animals were decapitated for further neurochemical assays and neurodegenerative analysis as depicted in Figure 1.

### 2.6 EEG Acquisition

The 24 animals with electrodes were divided into groups in the same way as explained before and were administered their respective treatments for 21 days with PTZ 40 mg/kg on alternate days (11 injections). EEG was recorded on the 1st, 5th, and 11th PTZ injections. Animals were connected to eight‐channel bioamplifiers (ADInstruments Ltd., Sydney, Australia) and an analog‐digital converter (PowerLab 8/35, ADInstruments) using signal sampling rate of 200 Hz and bandpass filtered between 0.1 and 60 Hz, and were allowed to be acclimatized for 30 min [15]. PTZ 40 mg/kg was administered after 30 min of designated treatment. The EEG was recorded for 30 min after the injection of PTZ to correlate behavioral changes with electrographic aberrations in kindled versus non‐epileptic animals.

### 2.7 Open Field/Infrared Actimeter Test

Open field/infrared actimeter test (OFT) is a commonly used behavioral paradigm to assess the impact of therapeutic drugs on locomotive activity as well as exploratory and anxiety‐like behavior. The acclimatized animal was introduced into a square box of 45 × 45 × 20 cm dimensions (Panlab Harvard apparatus IR Actimeter, Spain) with 16 × 16 upper and lower infrared beams to monitor the horizontal & vertical movement of the animal. The animals were placed in this area and video was recorded for 5 min to check their locomotion in horizontal and vertical directions. The video was recorded via Logitech software and then analyzed by using AnyMaze video‐tracking software. For anxiolytic profiling, the whole area was divided into two zones: one is the central zone and the other is the peripheral zone [17].

### 2.8 Light and Dark Test (L/D)

The light and dark (L/D) transition test is handy as it requires no prior training sessions and is a reliable test to evaluate anxiety‐like behavior in experimental rodents. The test is based upon the natural preference of mice toward the enclosed dark arenas over the aversive light area [18]. For assessment of anxiolytic behavior, each animal was introduced into the lit zone (21 × 21 × 25 cm) with black acrylic inserts used to separate the dark and light zone (20 × 40 × 40 cm) conjoined by a small opening (7 × 6 cm). Animal's investigation time and number of entries were noted for the 5 min to demonstrate variance in anxiety‐like behavior between the epileptic and non‐epileptic group.

### 2.9 Y‐Maze Test

The short‐term working memory in mice was measured in a trio‐arm y‐shaped maze (40 × 8 × 15 cm) by noticing the exploring pattern for the total span of 5 min, as animals have to recall the previously visited arm, and mice having increased percentage alternation depicts good memory [17]. The formula used to measure spontaneous alteration is as follows: Spontaneous alternations % (SAP) = [no of alterations/total arm entries‐2] × 100.

### 2.10 Novel Object Recognition Test

The novel object recognition (NOR) test is commonly employed to measure several phenotypic aspects of short‐term recognition memory and was performed in two sessions named acquisition and trial. During training, two identical objects were introduced into the square box (40 × 40 cm) with walls high enough to prevent the animal's escape from the box (38 cm), and mice were allowed to traverse two identical objects for the total duration of 5 min. During the trial session, one of the previously familiar objects was replaced with an unknown novel object and allowed to explore for another 5 min after a retention interval of 1 h to access short‐term working memory [19]. Mice with intact neurological function will spend more time exploring unknown novel object rather than the familiarized one. The object recognition index was calculated as per the formula: Discrimination index = (time with novel object—time with familiar object)/(time with novel object + time with familiar object).

### 2.11 Passive Avoidance Test

The passive avoidance (step through) test (PAT) is carried out to assess long‐term memory in rodents. The passive avoidance step through the apparatus consists of two compartments characterized as dark and light zones (24 × 20 × 20 cm) with an automated stainless steel door in between (Gemini avoidance system, San Diego Instruments, USA). This test is carried out in two sessions. One is the training session, that is, rodents were placed in the lighted area for acclimatization for almost 30 s then the door was opened and animals remained in the dark area for almost 150 s. An electrical shock of 0.5 mA was given to the animal in the dark area for almost 2 s. The other session is the test session after 24 h of the training. At first, the rodents were acclimatized to the light area for 20 s, then the door was opened and the rodents' latency to enter the dark area was observed for 300 s without shock delivery. This test evaluated long‐term memory retention in rodents after 24 h. The animals with intact memory will show longer escape latencies and preference toward the lit zone [20, 21]. All data regarding escape latencies were generated by Gemini Software Version 1.0.4.

### 2.12 Morris Water Maze Test

This test examines the characteristics of long‐term spatial memory in animals by tracing their ability to locate hidden rescue platform in a water‐filled maze opacified with the help of some non‐toxic dye [22]. The apparatus used in this test consists of a water‐filled tank almost 100 × 60 cm in length and width. A square platform of almost 12 × 12 cm was placed in the middle of the southwest pole. Furthermore, proximal and distal orientation cues of different colors and geometrical shapes were placed on the interior as well as the exterior side of the water maze. This test was carried out in three sessions namely acquisition, trial, and probe. During the acquisition phase, mice were given three trials per day with a cutoff latency of 120 s. Animals that were unable to navigate the platform within 2 min were gently propelled toward the visible platform and permitted to stay there for 10 s to acclimatize with orientation cues. In the experimental phase, the cue‐associated learning of animals was assessed by observing their ability to find a submerged platform (1 cm below the water) for 120 s for 3 days. On the 6th day, the platform was removed and the memory consolidation of animals was noticed by observing their latency to enter SW (southwest) zone, entries, and time spent in the target quadrant.

### 2.13 Sucrose Splash Test

This test is used to test the depressive‐like behavior in animals. In sucrose splash test (SST), 10% sucrose solution is sprayed over the dorsal top/back of the animals which makes the fur untidy. The dirty fur reinforces the mice to spontaneously start self‐grooming behavior. The grooming behavior of these animals was observed via 5 min video recording. Animals were observed for the grooming/motivational behavior of their nose, face, head, and body. Grooming frequency and grooming latency were the parameters used to evaluate self‐care behavior [23].

### 2.14 Brain Extraction and Homogenization for Biochemical Analysis

After the completion of behavior assays, animals were sacrificed for brain isolation (*n* = 3). These extracted brains were then homogenized separately in PBS solution (pH 7.4, Solarbio, Life Sciences) followed by centrifugation for 10 min at 12,000 rcf for a total span of 10 min at a controlled and constant temperature of 4°C [24]. The pellet was discarded and clear supernatants were carefully stored at −40°C with proper sample labels to assess neurochemical alterations to elucidate the protective impact of dual therapy with BRV 10 + PRO 1 in PTZ kindled mice. Four markers of oxidative stress, malondialdehyde (MDA), catalase (CAT), superoxide dismutase (SOD), and acetylcholinesterase (AchE) were evaluated as per the previously reported protocol and detail has been provided in Appendix S1 [19, 25]. Protein quantification was commenced for each brain sample as per Lowry's method [26].

### 2.15 Histopathological Analysis

For histopathologic analysis, formalin‐fixed (4% in PBS), paraffin‐embedded mouse brain sections (5 μm thickness; *n* = 3) were deparaffinized in xylene, rehydrated with alcohol in a series, rinsed with tap and distilled water, and stained with 0.1% cresyl violet (Alfa Aesar, Thermo Fischer Scientific, USA) at 50°C for 20 min. Sections were then rinsed with distilled water, differentiated in 95% ethanol, washed with absolute alcohol, cleared in xylene, and mounted with DPX medium (Sigma‐Aldrich) and a coverslip. For the assessment of PTZ‐induced hippocampal neurodegenerative changes and the subsequent effect of therapy with probiotics and BRV intervention, prepared brain tissue slides were then visualized, and the region of interest was located on an Olympus CH‐2, 838388 microscope, Japan. Photomicrographs were acquired at 4×, 10×, and 40× magnification using an Omax digital camera and ToupView software.

### 2.16 Statistical Evaluation

GraphPad prism v.8 for Windows (GraphPad Software, San Diego, CA, USA) was used for statistical evaluation of data. Prior to any data evaluation, data normality was computed using Shapiro–Wilk and Kolmogorov–Smirnov test. One‐way ANOVA followed by post hoc Dunnett's multiple comparisons was used to detect variance between PTZ‐kindled and treated animals. Repeated measure two‐way ANOVA followed by Dunnett's multiple comparison was utilized for seizure scoring and mean escape latencies in the Morris water maze (MWM) test. All data values are presented as mean ± SEM. A *p* < 0.05 was considered statistically significant.

## 3 Results

### 3.1 Effect of Probiotics and BRV Alone and in Combination Therapy on PTZ Kindling Progression

**FIGURE 2:**
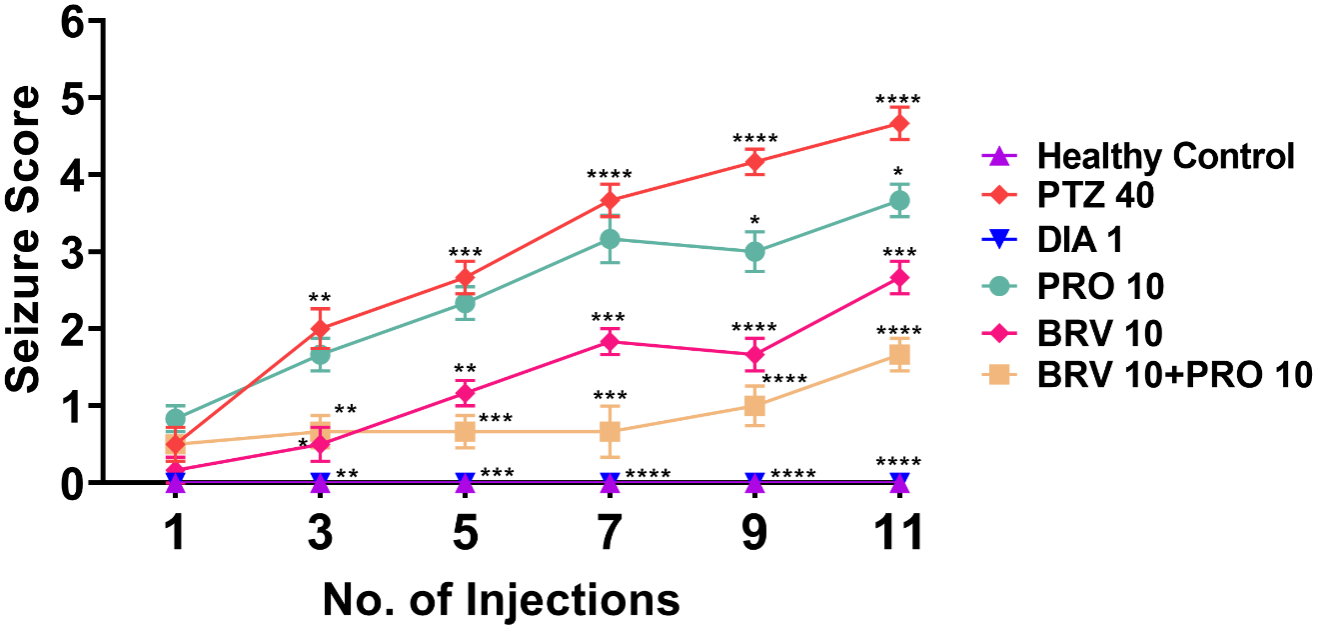
Graphical presentation of the impact of chronic probiotic supplementation alone and in combination with BRV on seizure score assessed as per Racine scale during the PTZ‐kindling process. The kindling was carried out by injecting PTZ (40 mg/kg) on alternate days for a total of 11 injections to all animals except healthy control mice. The results were evaluated by applying repeated‐measure two‐way ANOVA followed by Dunnet's test revealing the significant intergroup difference when compared with the PTZ group. The values are shown as the mean ± SEM (*n* = 6), while *p < 0.05, ***p < 0.001, and ****p < 0.0001 represents statistical significance.

Brain insults triggered via repetitive injection of chemoconvulsant PTZ at the subthreshold dose of 40 mg/kg in an alternate fashion for 3 weeks resulted in pulsating epileptic response as animals showed gradual escalation in seizure severity from mild partial seizures to severe generalized convulsions (*F* [5, 150] = 225.8, *p* < 0.0001). Almost, every mouse in PTZ kindled group showed Stage 3 myoclonus during the 5th–6th stimulation. With further PTZ‐instigated brain insult, these animals showed Stage 4 rearing by the 7th stimulation which then culminates into generalized tonic–clonic convulsions (GTCS) by the 8th–11th stimulation resulting in fully‐kindled mice ruminating clinical epilepsy. The mice treated with diazepam 1 mg/kg as a standard drug showed complete protection against PTZ‐provoked epileptic seizures. The animals receiving PRO 10 as a monotherapy depicted a slight increase in convulsive threshold by the 9th–11th stimulation but still 80% of the animals presented Stage 4 seizures on the 21st day. In contrast, therapy with BRV 10 elicits protection from rearing and GTCS as almost most of the animals showed Stage 3 forelimb clonus. Combination therapy with BRV 10 + PRO 10 robustly halted kindling progression and improved behavioral outcomes as animals depicted only partial seizures of Stage 2 by the 9th–11th stimulation as compared to epileptic rats as shown in Figure 2.

**FIGURE 3:**
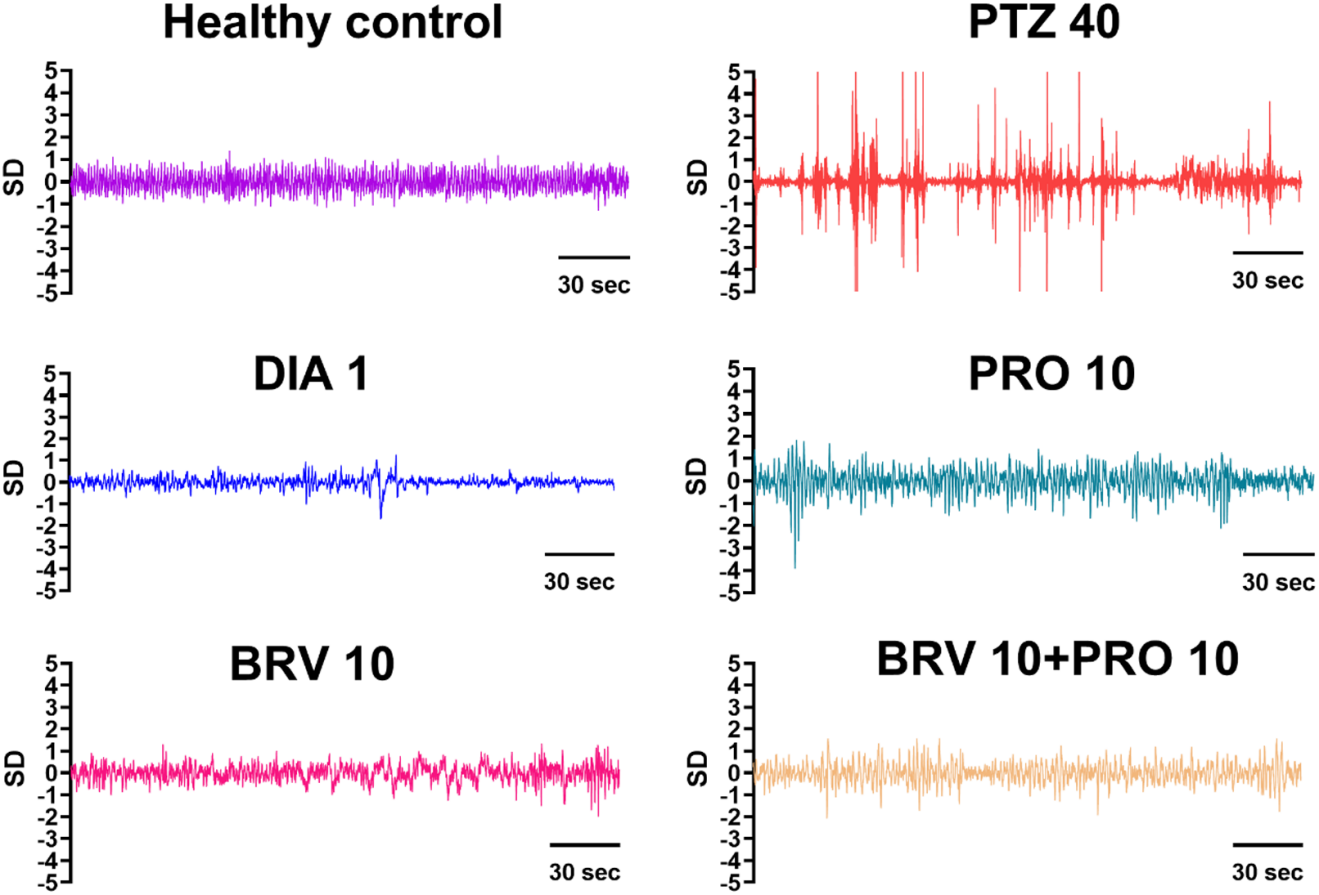
Magnified electrograms presenting the impact of probiotics alone and in combination with BRV on the progression of epileptogenic foci. The animals were given their respective pretreatment with PRO 10 mL/kg and BRV 10 mg/kg alone and in combination and after post 30 min after therapy, they were challenged with PTZ 40 mg/kg. The EEG was recorded for 30 min after the administration of PTZ. The tracings represent ictal discharges in PTZ‐kindled mice during Stages 4–5 seizures; however, the high voltage spiking activity was reduced in combination therapy with BRV 10 + PRO 10.

Furthermore, in mice with cortical electrode implants, vEEG was recorded for post 30 min after the first, fifth, and eleventh PTZ injection. As illustrated in Figure 3, the vEEG recordings unveiled that PTZ instigated aberrant electrographic changes which were in accordance with the observed behavioral averments in diseased and monotherapy versus combination groups. The magnified electrogram showed that PTZ‐treated mice presented high‐grade epileptiform ictal discharges with increased amplitude and frequency followed by reduced brain activity in the post‐ictal phase as the animal resumed baseline behavior. The electrographic seizures were not observed in the diazepam‐treated group. The abnormal rhythmic brain activity was noticed in PRO 10 and BRV 10 groups but the high amplitude ictal discharges, the hallmark of active epilepsy in PTZ kindled group were not observed by the 9th–11th stimulation in mice treated with BRV 10. Similarly, in the group treated with BRV 10 + PRO 10, most of the animals maintained baseline brain activity and a significant reduction in the progression of the epileptogenic process.

### 3.2 Open Field Activity

**FIGURE 4:**
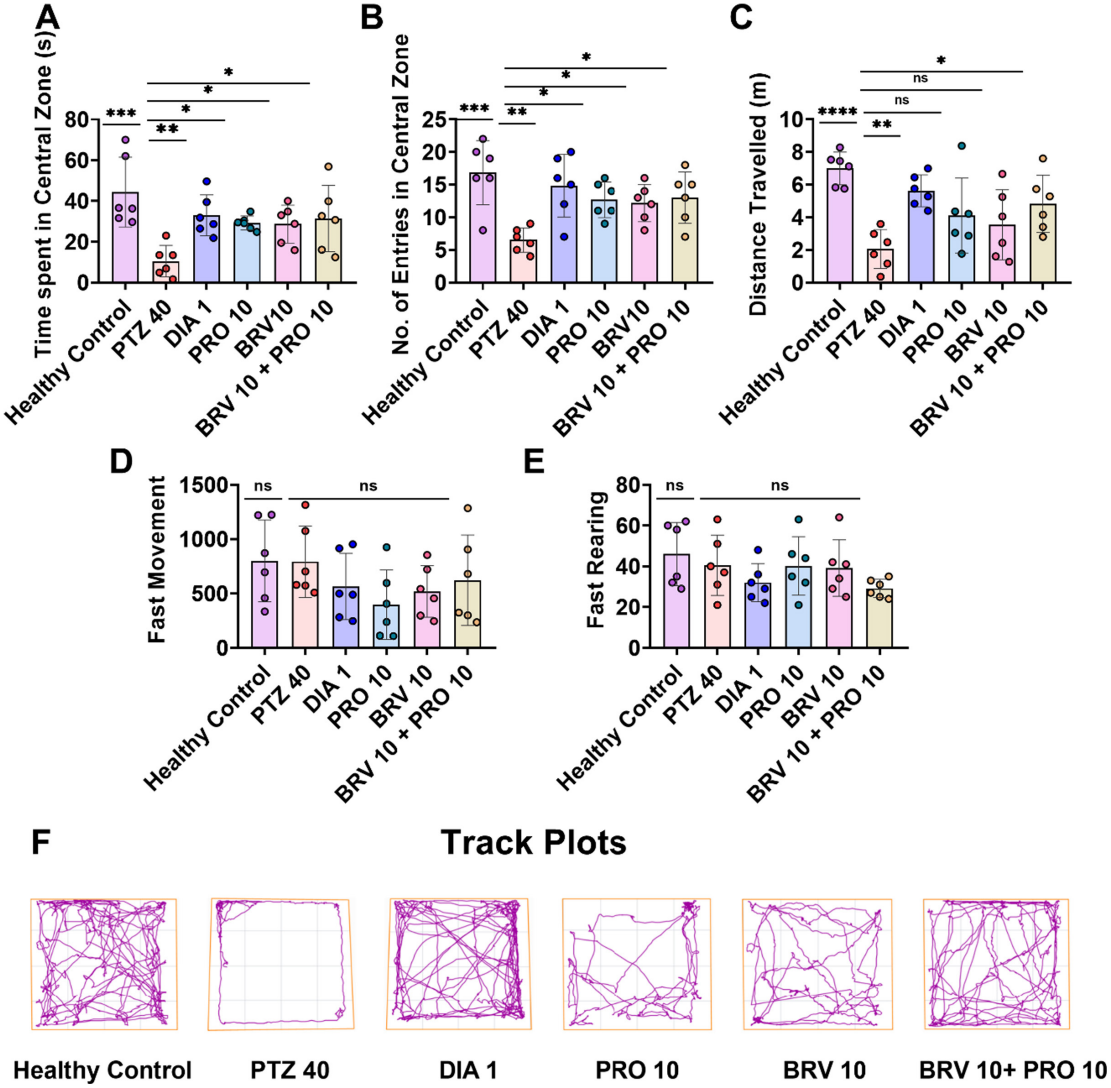
Evaluation of the preventive impact of PRO 10 mL/kg and BRV 10 mg/kg treatment alone and in combination on the anxiety‐like behavior and locomotive activity of mice assessed by OFT on the 22nd day of the post‐kindling process. (A) Time spent in the central zone, (B) number of entries in the central zone, (C) distance traveled, (D) fast movements, and (E) fast rearing. (F) Representative track plots depicting reduced risk‐taking behavior as animals continued to roam around peripheries as compared to treated mice. The results were analyzed by applying one‐way ANOVA followed by Dunnett's test in comparison with the PTZ group. The data are represented as a mean ± SEM (*n* = 6). *p < 0.05, **p < 0.01, ***p < 0.001 and ****p < 0.0001 representing statistical significance.

To evaluate the impact of gut microbiota alteration on PTZ kindling evoked anxiety‐like behavior, the animal's exploratory behavior in the open arena was assessed in terms of their innate predilection toward the central open area by observing their investigation time and number of visits in the central arena (*F* [5, 30] = 5.269, *p* < 0.0014) and (*F* [5, 30] = 5.316, *p* < 0.0013, respectively). The neurologically intact healthy animals represented reduced fear toward the central arena as the animals explored that particular zone more often with prolonged investigation time (44.36 ± 7.00 s) and number of visits (16.83 ± 2.00). In contrast, kindled mice depicted phenotypic anxiety‐like behavior with significantly reduced duration in the central zone (10.53 ± 3.15 s; *p* < 0.0001) as well as number of entries (6.50 ± 0.76; *p* = 0.0002) as shown in Figure 4A,B. The kindling‐associated anxiety‐like behavior was alleviated by monotherapy with PRO 10 and BRV 10 administration, but the combination therapy exerted much beneficial effects on anxiety‐like psychiatric signs as evidenced by increased duration of stay (31.43 ± 6.63; *p* = 0.0176) and the number of entries (13.00 ± 1.61; *p* = 0.0201) in comparison to epileptic animals yielding results comparable to DIA 1 standard treatment.

Furthermore, the gross locomotive activity of animals was also evaluated by determining the distance traveled in the open field arena. The intergroup variability was noted for distance traveled (*F* [5, 30] = 6.581, *p* < 0.0003). Healthy animals to some extent traveled more distance (6.91 ± 0.41 m) as compared to PTZ 40 group (2.04 ± 0.48 m; *p* < 0.0001) which presented a shorter distance traveled. Concomitant therapy with BRV 10 and PRO 10 resulted in a longer distance traveled (4.82 ± 0.71; *p* = 0.0276) as compared to single therapy and PTZ kindled group (Figure 4C).

Moreover, fast movements (vertical movements) and fast rearings (horizontal movements) were observed in the IR actimeter test to determine the impact of drug interventions on spontaneous movements. Both parameters were found to be non‐significantly different among all groups with (*F* [5, 30] = 1.337, *p* < 0.2759) and (*F* [5, 30] = 1.420, *p* < 0.2457), respectively as presented in Figure 4D,E. The healthy group showed fast movements (800.83 ± 153.52) and fast rearing (46.00 ± 6.33) in the span of 5 min which were slightly greater than epileptic animals (792.50 ± 133.80 and 40.50 ± 6.05; *p* = 0.9101, respectively). However, daily supplementation of BRV 10 + PRO 10 resulted in a non‐significant decrease in both fast movement (621.83 ± 169.88; *p* = 0.8453) and fast rearings (29.16 ± 1.83; *p* = 0.4072).

### 3.3 Anxiolytic Effects in L/D Test

**FIGURE 5:**
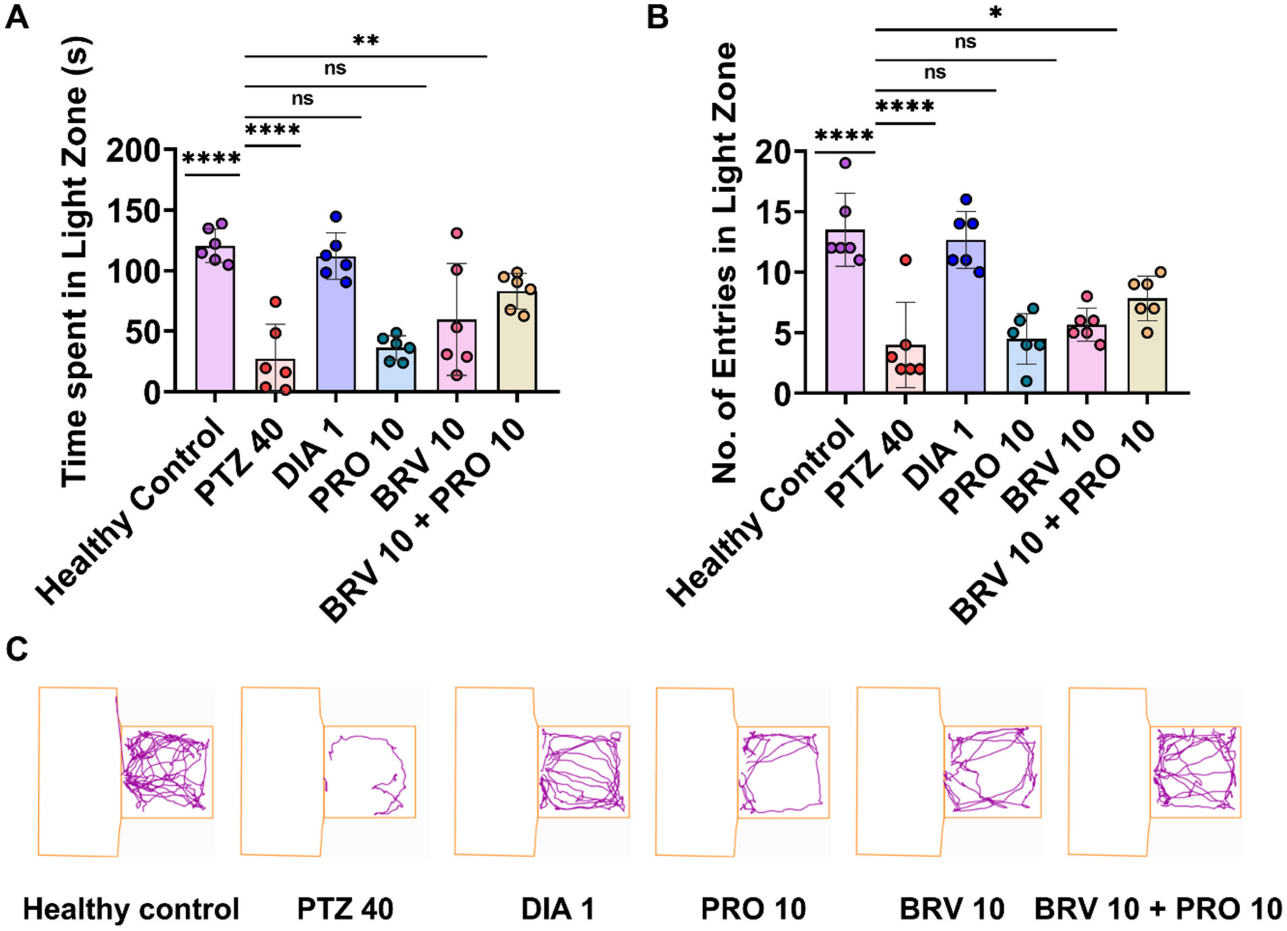
Anxiolytic profiling of combination therapy with BRV 10 + PRO 10 in L/D test. Mice were allowed to explore the light and dark zones for 5 min on the 23rd day. (A) Time spent in the light zone, (B) number of entries in the light zone. (C) Representative track plots showing the impact of BRV 10 + PRO 1 alone and in combination on anxiety‐like behavior and exploration of a novel environment. The outcomes were evaluated by applying one‐way ANOVA by comparing the results of all groups with the PTZ group. Data are presented as mean ± SEM (*n* = 6) while *p < 0.05, **p < 0.01, and ****p < 0.0001 showing statistically significant significance.

Light zone preference was noted to elucidate the anxiolytic impact of combinatorial therapy with BRV 10 + PRO 10. The significant intergroup variability was observed for the time spent and the number of entries in light zone (*F* [5, 30] = 9.634, *p* < 0.0001) and (*F* [5, 30] = 14.11, *p* < 0.0001) respectively as shown in Figure 5A,B. The kindled mice preferred darker compartment as evidenced by their reduced time spent (27.26 ± 11.63 s) and the number of entries (4.00 ± 1.43) with *p* < 0.0001 in a lit zone in comparison to healthy mice which showed greater preference toward the open aversive lit arena with increased exploration time (120.60 ± 5.65 s) and number of entries (13.50 ± 1.23). Combination therapy remarkably enhances risk‐taking behavior and exerts anxiolytic impact with increased duration of stay (83.03 ± 6.02 s; *p* = 0.0028) and number of visits (7.83 ± 0.74; *p* = 0.0462) into the light zone. However, monotherapy with PRO 10 and BRV 10 yielded non‐significant results.

### 3.4 Short‐Term Memory Assessment via Y‐Maze (Spontaneous Alternation) and NOR Test

**FIGURE 6:**
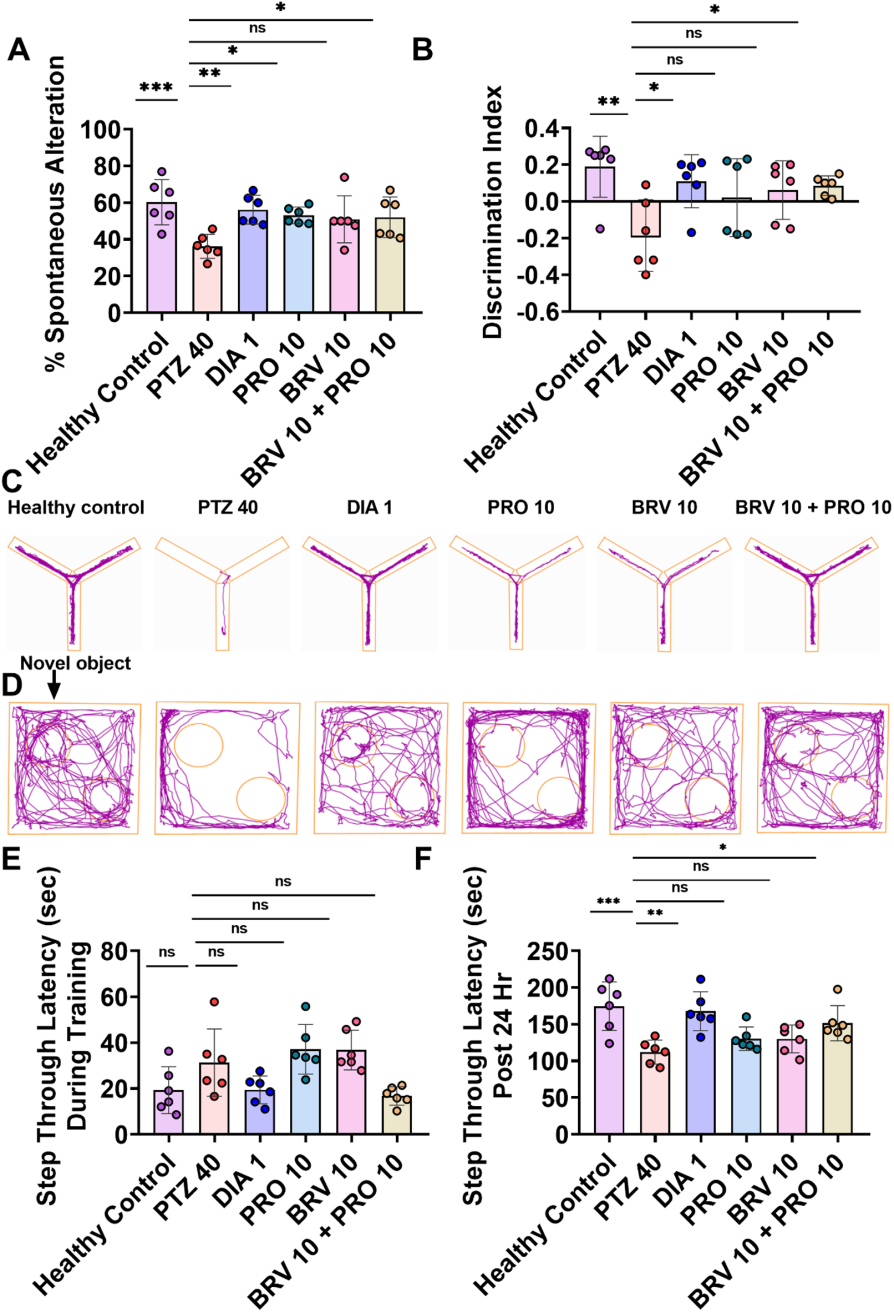
The impact of BRV 10 and PRO 10 alone and in combination on the learning and memory aspects of animals. On the 24th day, animals were tested for their short‐term memory in the *Y*‐maze test noted by observing their percentage spontaneous alteration. Animals were further tested for episodic memory in the NOR test on the 25th day by monitoring their interaction with novel and previously familiarized objects. (A) % spontaneous alteration, (B) discrimination index. (C) Representative track plots showing improved alternation behavior in mice treated with BRV 10 + PRO 10 combination regime. (D) Representative track plots illustrating the impact of combination therapy on PTZ‐induced short‐term memory deficits. Moreover by the 26th–27th day epileptic and treated animals were tested for long‐term recognition memory by noticing their escape latencies shock zone in the PA test. (E) Step through latency during training (s). (F) Step through latency post 24 hr (s).  One way‐ANOVA following Dunnett's test was performed to analyze the data representing as mean ± SD (*n* = 6) *p < 0.05, **p < 0.01, and ***p < 0.001, as statistically significant in comparison with the PTZ group.

To assess short‐term memory, mice were allowed to explore a trio‐arm y‐shaped maze, and their % SAP was noticed (*F* (5, 30) = 4.292, *p* = 0.0046). The diseased animals illustrated altered remembrance of a previously visited arm of the maze and their trajectory fields showed reduced mean alternation (36.26 ± 2.66) as compared to healthy mice (60.29 ± 5.02) with *p* = 0.0008 (Figure 6A). In comparison with epileptic control, BRV 10 + PRO 10 group showed exceptional improvement in cognitive capacity with increased alternation behavior (52.06 ± 4.51; *p* = 0.0339).

The results of the object recognition test demonstrated significant mean effects with the object discrimination index in the NOR test (*F* [5, 30] = 6.090, *p* = 0.0005). The test was commenced in two sessions; acquisition and trial and the discrimination index was computed during the trial phase. The healthy animals depicted no memory deficits as they spent more time exploring novel object rather than the previously investigated known object with improved recognition scores (0.28 ± 0.01) as compared to the PTZ 40 group (−0.18 ± 0.08) with *p* < 0.0001. However, the combination of BRV 10 + PRO 10 resulted in a noticeable improvement in the recognition index (0.08 ± 0.02; *p* = 0.0174) as animals interacted for longer duration with the unknown object as compared to epileptic control. Outcomes remained non‐significant for both BRV 10 and PR0 10 groups (Figure 6B).

### 3.5 Long‐Term Working Memory Assessment via PA and MWM Test

Statistically significant mean effects were noted for step‐through latencies in the PA test (*F* [5, 60] = 6.550, *p* = 0.0003). During the training session, no significant intergroup differences were noted as animals spontaneously entered into the shock compartment (dark zone) shortly after placing them into the lit chamber (Figure 6E). To evaluate long‐term working memory the animal's recalling capability was noted post‐shock at the timescale of 24 h. The outcomes of the test session after 24 h showed that kindled animals had poor memory consolidation of aversive stimuli as they left the lit compartment and entered the dark chamber (112.18 ± 6.59) in which previously they had received a shock as collated to healthy non‐epileptic rats (174.65 ± 13.52) with *p* = 0.0003. However, as compared to kindled epileptic mice, dual therapy with BRV 10 + PRO 10 significantly improved memory dysfunction as animals depicted increased aversion toward dark chamber (151.56 ± 6.80; *p* = 0.0264) as shown in Figure 6F.

**FIGURE 7:**
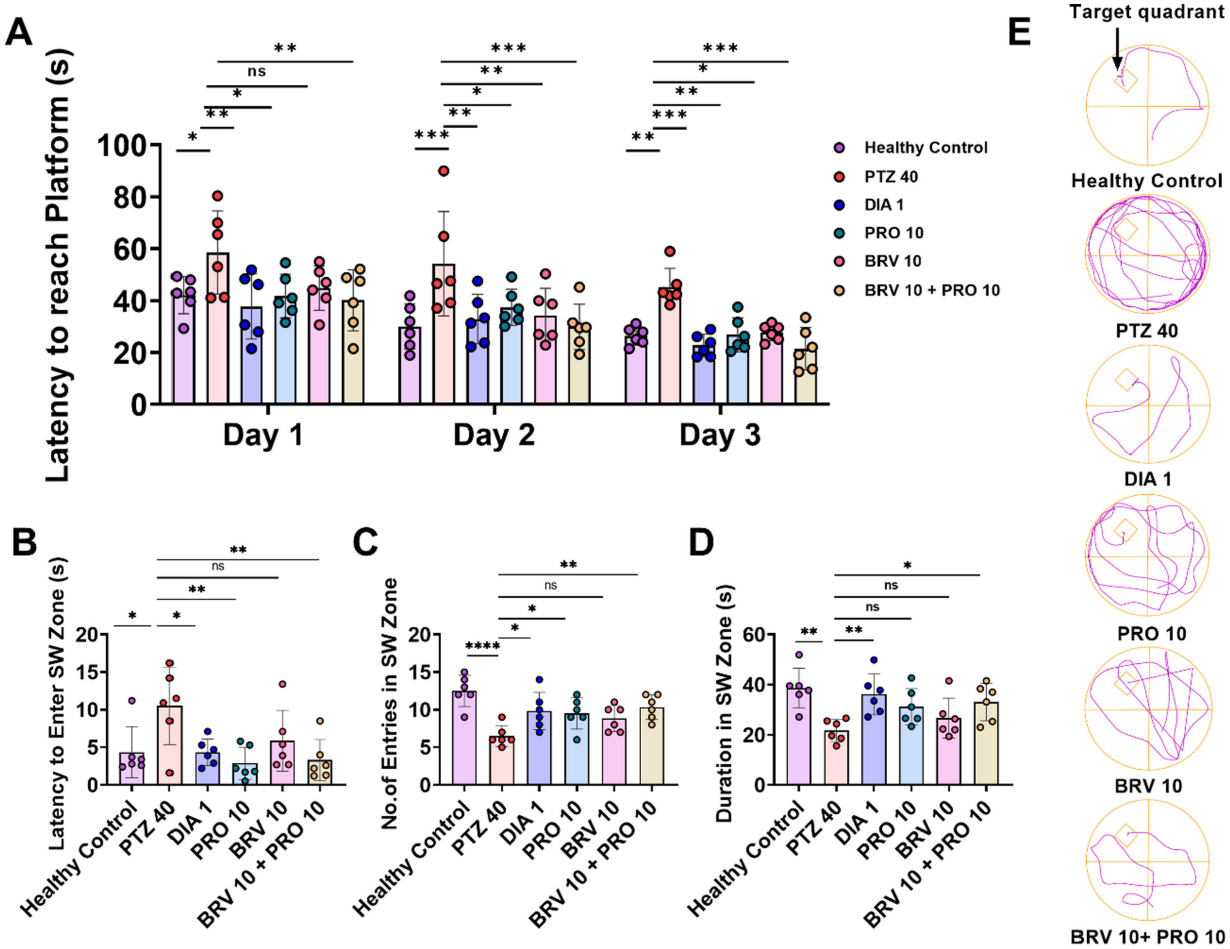
The impact of BRV 10 + PRO 10 alone and in combination on the long‐term spatial memory was assessed via MWM test from the 28th–33rd day. (A) Escape latencies on the testing days after training for initial 2 days with visible platform, (B) latency to enter the SW zone on probe day, (C) number of entries in the SW zone, (D) duration spent in the SWzone. (E) Track plots representing animal's learning aptitude and performance during experimental trials. Trajectory patterns unveiled positive impact of combinatorial regime of BRV 10 + PRO 10 to navigate hidden rescue platform with shorter escape latencies as compared to PTZ 40. Repeated two‐way ANOVA was applied for the escape latencies on the test days, while the other parameters of MWM were analyzed by applying one‐way ANOVA followed by Dunnett's test by comparing with the PTZ group. Data are presented as mean ± SEM (*n* = 6) while *p < 0.05, **p < 0.01, ***p < 0.001, and ****p < 0.0001 were considered statistically significant.

Cue‐associated spatial memory and learning were measured in PTZ‐kindled and treated mice which revealed significant intergroup differences for escape latencies (*F* [5, 90] = 12.59, *p* < 0.0001). By the 3rd–5th day during the experimental session with the submerged rescue platform, the epileptic mice depicted persistent thigmotaxic behavior and were unable to navigate the platform situated in the SW zone of the water maze. On 3rd day, PTZ‐kindled animal mice depicted longer escape latencies (58.56 ± 6.55 s) as compared to the healthy group (42.10 ± 2.93) with *p* = 0.0209. In contrast healthy control further illustrated improved learning capacity as they took a shorter time to locate the hidden platform on the 4th and 5th day of the experimental session with escape latency of 30.05 ± 3.53 and 26.40 ± 1.43, respectively. PTZ control animals still depicted poor learning as evidenced by their time spent in finding the rescue podium on the 4th (54.21 ± 8.19 s; *p* = 0.0003) and 5th day (45.18 ± 2.95; *p* = 0.0064) as compared to healthy mice. The combination therapy with BRV 10 + PRO 10 reversed PTZ‐instigated memory deficits following the inception of epileptogenic foci as animals reached on platform in 21.45 ± 3.31 s; *p* = 0.0004 on the 5th day, and the outcomes were comparable with the DIA 1 group (*p* = 0.0008) as depicted in Figure 7A.

Furthermore, in the probe trial, the animals demonstrated exceptional variation in latency to enter the SW zone (*F* [5, 30] = 4.027, *p* = 0.0065), number of entries (*F* [5, 30] = 6.202, *p* = 0.0005), and duration of stay (*F* [5, 30] = 4.338, *p* = 0.0043). The animals in the healthy non‐epileptic group readily entered into the target quadrant at 4.35 ± 1.38 s depicting enhanced learning and memory recall (Figure 7B). Moreover, on probe day healthy control animals entered the quadrant more frequently (12.00 ± 0.87) and spent more time in navigation of SW zone (38.65 ± 3.25 s). The diseased mice showed phenotypic memory deficits with longer latency to enter into the target zone (10.48 ± 2.08; *p* = 0.0167) as well as reduced number of entries (6.50 ± 0.56; *p* < 0.0001) and duration of stay in the zone (21.73 ± 1.79 s; *p* = 0.0018) in which platform was previously placed as illustrated in Figure 7C,D. The administration of the BRV 10 + PRO 10 combination for 21 days significantly attenuated kindling‐induced memory dysfunction as animals illustrated shorter latency to enter into the SW zone (3.30 ± 1.12; *p* = 0.0043). Moreover, they also presented an increased number of visits (10.33 ± 0.66; *p* = 0.0075) and spun there for longer duration (33.05 ± 3.03 s; *p* = 0.0480) indicative of their well‐preserved long‐term memory.

### 3.6 Self‐Care Behavior Assessed via SST

**FIGURE 8:**
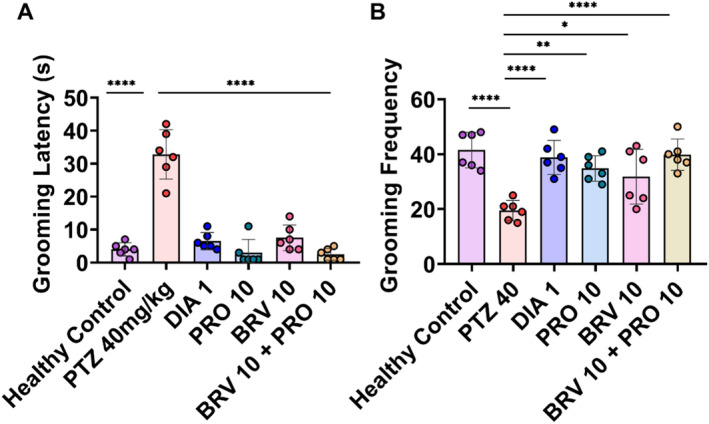
Assessment of self‐care behavior via splash test. On the 34th day animal's behavioral despair was evaluated by splashing 10% sucrose solution on the back of epileptic and treated mice. (A) Grooming latency (s). (B) Grooming frequency. One way‐ANOVA following Dunnett's test was performed to analyze the data representing mean ± SEM (*n* = 6), *p < 0.05, **p < 0.01, and ****p < 0.0001 as statistically significant in comparison with the PTZ group.

Self‐care behavior was evaluated in the sucrose splash test in which depressive‐like behavioral despair is characterized by reduced time spent in grooming or increased grooming latency (*F* [5, 60] = 48.55; *p* < 0.0001) as well as mean decrease in grooming frequency (9.562; *p* < 0.0001) indicating altered self‐motivation or apathetic behavior. PTZ‐treated animals showed a depressive state as evidenced by reduced motivational behavior (32.83 ± 3.04) and grooming frequency (19.50 ± 1.50) with *p* < 0.0001. In contrast, healthy animals showed normal behavior evidenced by reduced grooming latency (4.00 ± 0.18) and frequency (41.50 ± 2.64). Monotherapy with PRO 10 significantly altered stress‐mediated response, that is, depression probably via maintenance of a healthy intestinal biome and animals showed improved grooming behavior with decreased latency (3.00 ± 1.63) and escalated grooming frequency (34.83 ± 1.90; *p* = 0.0012). Furthermore, BRV 10 also preserved self‐care behavior to some extent with reduced grooming latency (7.50 ± 1.58) and a significant increase in grooming frequency (31.83 ± 4.06; *p* = 0.0101). The outcomes of combination therapy further reckon the protective impact of adjunct probiotic treatment with BRV as animals showed remarkable amelioration in depressive‐like behavior with significantly reduced grooming latency (2.50 ± 0.71) and substantial increase in grooming frequency (39.83 ± 2.33) with *p* < 0.0001 as demonstrated in Figure 8A,B.

### 3.7 Neurochemical Analysis

Significant mean effects were noted for the MDA (*F* [5, 12] = 7.229; *p* = 0.0025), a prestigious marker of lipid peroxidation, endogenous antioxidants, that is, catalase (*F* [5, 12] = 4.587; *p* = 0.0143) and SOD (*F* [5, 12] = 4.482; *p* = 0.0155). MDA levels were found to be approximately twofold higher in the kindled brains as compared to normal healthy brains with *p* = 0.0099. These altered levels were efficiently alleviated by the BRV 10 + PRO 10 combination as compared to the protective impact of monotherapy (*p* = 0.0011). Furthermore, evaluation of antioxidant capacity revealed significantly reduced catalase and SOD activity in PTZ control insulted brains in comparison to healthy brains with *p* = 0.0042 and *p* = 0.0050, respectively. BRV 10 + PRO 10 restored imbalanced physiological antioxidant levels, thus preventing further brain injury as evidenced by significantly increased levels of catalase (*p* = 0.0490) and SOD (*p* = 0.0346) (Table 1).

**TABLE 1:** Post‐kindling neurochemical assessment of whole brain tissue extracted on the 35th day after sucrose splash test. One‐way ANOVA was applied to assess the MDA, catalase, and SOD, levels followed by Dunnett's test comparing the outcomes with the PTZ group.

Groups	MDA (nmol/mg/protein)	Catalase (μmol/min/mg/protein)	SOD (units/mg/protein)
Healthy control	288.77 ± 18.35	569.01 ± 24.58	192.07 ± 2.79
PTZ 40	533.62 ± 93.25**	216.91 ± 34.71**	96.26 ± 6.24**
DIA 1	335.89 ± 21.65*	539.92 ± 65.47**	185.12 ± 13.46**
PRO 10	320.67 ± 24.94*	443.63 ± 98.18^ns^	158.55 ± 10.11^ns^
BRV 10	476.31 ± 30.84^ns^	438.51 ± 40.46^ns^	154.27 ± 22.65^ns^
BRV 10 + PRO 10	206.54 ± 35.75**	454.83 ± 51.46*	167.03 ± 26.63*

*Note:* All data values are presented as mean ± SEM (*n* = 3) while *p < 0.05 and **p < 0.001 present statistical significance while ns represents non‐significant outcomes.

### 3.8 Histopathologic Analysis Results

**FIGURE 9:**
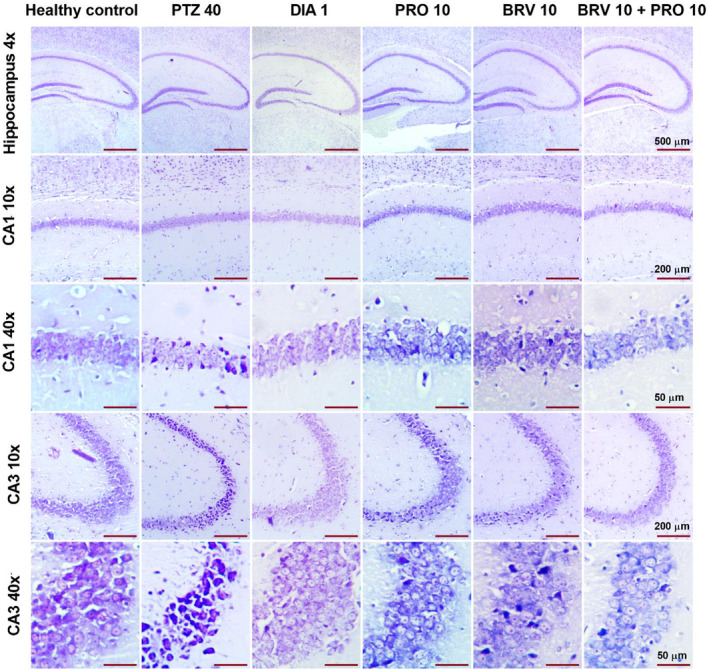
Digital photomicrographs of Nissl's stained cornu‐ammonis (CA1 and CA3) represent the neuroprotective effect of BRV 10 + PRO 10 on PTZ‐induced neurodegeneration. Scale bar = 500 μm at 4×, 200 μm at 10×, and 50 μm at 40× magnification (*n* = 3).

Chronic changes in cornu ammonis cellular pathology, that is, neuroinflammation and cell death after exposure of mice to PTZ‐induced chemical kindling and neuroprotective potential of subsequent treatment with probiotics in combination with BRV was evaluated using cresyl violet staining. Brain sections of the healthy control demonstrated intact cellular morphology in both regions CA1 and CA3 of the hippocampus with preserved cytoarchitecture. However, repetitive PTZ administration elicits nuclei fragmentation, pyknosis (oval‐shaped condensed nuclei appearing as darkly stained cells), tissue disruption, and cytoplasmic vacuolation with depleted neuronal density. In the brains of animals treated with DIA 1, the visual field showed clear neuronal cells and an absence of apoptotic bodies. Monotherapy with PRO 10 and BRV 10 to some extent depicted loss of integrity, hyperchromatic cells, maculation, and shrunken cytoplasm in both CA1 and CA3 regions. Combination therapy with BRV 10 + PRO 10 ameliorated these epileptogenic prompted degenerating pathologic alterations as animal brains showed fewer pyknotic cells in CA1 and CA3 regions as demonstrated in Figure 9.

## 4 Discussion

The recent research in the field of epilepsy has unraveled the plausible preventive role of reconstructing the gut microbiota via non‐pharmacological interventions such as ketogenic diet (KD), synbiotics (mixture of pro and prebiotics), and fecal microbiota transplants in drug‐resistant epilepsy [27]. In intractable epilepsy, the gut microbiome composition is characterized by abridged diversity and increased proinflammatory bacteria leading to poor prognosis. Therefore, In the current study, we investigated the regulatory influence of the 21‐day treatment of probiotic supplementation as an add‐on therapy with BRV, an SV2A modulator at the dose of 10 mL/kg and 10 mg/kg, respectively on the progression of the epileptogenic process, associated electrophysiological and behavioral arrests, redox stress and neurodegeneration in an animal model of PTZ‐induced generalized epilepsy.

Our outcomes in the current study highlighted that long‐term 21‐day probiotic supplementation along with BRV substantially halted epileptogenic brain activity by retarding the development of both whole‐body myoclonus and GTCS. This intervention depicted a favorable impact in preserving baseline rhythmic brain activity in animals challenged with PTZ and this effect was also supported by reduced seizure score as per Racine's scale and the percentage of animals that can be categorized as fully kindled as compared to epileptic animals which depicted high amplitude epileptiform discharges during Stage 4–5 seizures. Electrographic profiling favors combination therapy over monotherapy, as evidenced by the augmented epileptic response by the end of the 11th PTZ injection. Several strains of microorganisms can procreate crucial inhibitory neurotransmitter for regulating seizure threshold GABA, in which Lactobacilli and *Bifidobacteria* are identified as the main producers of GABA as these commensal species possesses the key catalyzing enzymes glutamic acid decarboxylase (GAD) to generate GABA from its precursor monosodium glutamate (MSG) which usually acts locally via enteric nervous system [28]. However, acetate, a precursor of GABA synthesized in the colon via gut microbiome enters the BBB after crossing intestinal barrier and affects neuroglial GABAergic cycling pathways where it is permuted into glutamine and traversed back to neural cells. Glutaminase prompts conversion of glutamine to glutamate, which is shunted back into GABA cycle. Thus, there is accumulating evidence of a plausible role of intestinal dysbiosis in the alteration of physiological GABA balance and increase in glutamatergic neurotransmission owing to dysregulated GABA/glutamate ratios in the brains [29]. Furthermore, an imbalance in intestinal flora also triggers immune responses which can propagate CNS injury and probiotics can promote this balance toward an anti‐inflammatory phenotype reducing neuroinflammation, an igniting factor in the pathogenesis of epileptogenic brain rewiring further supporting the role of probiotics as a potential preventive strategy for refractory epilepsy [30]. These aforementioned mechanisms might have exerted beneficial influence on the health and behavior of animals subjected to PTZ‐induced chemical kindling.

Pathologies related to gut dysbiosis are also linked to altered behavior, cognition, mood, and stress response [31]. PTZ kindling is an etiological platform that can mimic a wide range of epilepsy‐prompted behavioral, neuropsychiatric, and neurochemical repercussions. As active epilepsy is associated with a high incidence of comorbid psychiatric disorders, thus it is crucial to have novel antiepileptic therapeutic paths that positively target epileptogenesis‐induced neurophysiological disturbances.

HPA axis dysregulation, a main component of stress response is strongly linked with the progression of mental disorders such as anxiety. Stress‐induced alterations in the gut microbiome can lead to activation of the HPA axis which in turn can hike cortisol secretion [27]. Gut dysbiosis can promote anxiety disorder both by neurological and systemic inflammation, that is, increased gut permeability, also a key activator of immune response, as it allows passage of endotoxins (lipopolysaccharides) into the bloodstream. Moreover, peripheral input can upregulate the expression of neurotoxins, and inhibit neurotransmitters which negatively influence feelings of apprehension. Substantial surges in the levels of proinflammatory cytokines, reduced SCFAs, and downregulation of anti‐inflammatory signaling pathways have been described in patients clinically diagnosed with anxiety disorder as compared to healthy individuals, thus validating the link between inflammation and anxiety [32]. Serotonin is another important neurotransmitter involved in the regulation of mood and anxiety. Biometabolism of tryptophan via the kynurenine pathway generates key pathophysiological metabolites of anxiety. It is commonly accepted that indoleamine‐2,3‐dioxygenase which is shunted into the kynurenine pathway is activated by the increase in pro‐inflammatory cytokines highlighting the psychoimmunological mechanistic insights of anxiety [33]. Restoration of microbiome diversity via probiotic supplementation can suppress unhinged inflammation in epileptic patients with comorbid anxiety as it can impact serotonin production via kynurenine and tryptophan metabolism. Neurobehavioral analysis of chemically kindled mice demonstrated a marked increase in anxiety‐like behavior as compared to naïve‐healthy control. Results of OFT and L/D validated that chronic adjunctive probiotic supplementation (10 mL/kg) with BRV (10 mg/kg) did not negatively impact the animal's spontaneous exploratory behavior and at this dose, no CNS depressant effects were noted. BRV 10 + PRO 10 dual regime significantly improved anxiety‐like phenotype as animals fearlessly investigated aversive open and lit arena in OFT and L/D tests, respectively.

Cognitive perturbations in epilepsy result from seizure‐driven injury to neural networks involved in memory consolidation and data processing. Further cornu ammonis sclerosis, atrophy, and decrease in neuronal density, neuronal inflammation are the characteristic features of both partial and generalized epilepsy [34]. Although, there are plenty of clinical and preclinical studies suggesting a relationship between intestinal microbiota and normal brain function. However, preclinical data elucidating the direct impact of chronic supplementation of probiotics in improving epilepsy‐induced memory and learning impairments is scarce. Previously, Gareau et al. [35] published that gut dysbiosis in GF (germ‐free animals having no microbiota), enteric pathogen‐mediated bacterial infection, and probiotic supplementation all can modify learning aptitude and cognitive consolidation capacity. Our outcomes suggested severe impairments in both short‐term and long‐term spatial memory in PTZ kindled which were exceptionally ameliorated by BRV 10 + PRO 10 treatment hinting at the potential modulatory effect of SV2A glycoprotein on restoration of synaptic dysregulation and depressed long‐term potentiation. Probiotics on the other hand decrease inflammation preventing cell loss and aberrant structural alterations resulting in cognitive disturbances.

Depression is one of the most prevalent comorbidities in patients with epilepsy (PWE). Neurological structure alterations, changes in neurotransmitter release, inflammation, genetic and inflammatory factors all can modulate the development of comorbid depression and enhanced seizure susceptibility in epilepsy [36]. According to a recent report, decreased abundance of *Lactobacillus*, *Lachnospiraceae*, *Firmicutes*, and *Bifidobacterium* in depressed patients [37]. Negative changes in microbiome diversity and taxonomic abundance can promote depression by increased intestinal barrier permeability, systemic inflammation, changes in tryptophan biometabolism as well as changes in brain‐derived neurotrophic factor (BDNF). A leaky gut can allow microbial products such as LPS to trigger inflammatory reactions via proinflammatory cytokines that can cross the BBB leading to disrupted brain activity implicated in major depressive disorder via modulation of BDNF, kynurenine, and serotonin signaling pathways [38]. Our outcomes demonstrated the mood‐stabilizing impact of probiotic administration with BRV as treated mice showed reduced grooming latency and increased number of groomings in splash test with the mean effects to be more statistically significant in the group receiving combination intervention as compared to single therapy. In comparison with treatment, PTZ‐kindled mice showed behavioral despair with a lack of self‐care behavior. However, drifts in the normal microbiome may correlate with altered mood and behavior, establishing a causal relationship between the microbiome and depression remains challenging, especially given the complex etiology of psychiatric diseases.

A growing body of literature supports the link between elevated redox stress, brain injury, and pathophysiogenesis of neurodegenerative disorders entailing epilepsy as well. Impairments in redox homeostasis, reduced endogenous antioxidant factors, and seizure chronicity play an essential role in prompting neuronal death [39]. Moreover, it is also suggested that reduced antioxidant capacity and an increase in by‐products of lipid peroxidation are some of the reasons behind abnormal neuronal synchrony. Therefore, agents that can restore baseline oxidant/antioxidant levels have been expected to improve the prognosis of epilepsy. In the current study, we also deciphered whether the probiotic bacterial supplementation can underlie brain levels of oxidative stressor MDA. Moreover, we also determined antioxidant potential by determining activity levels of SOD and catalase in kindled and treated rats. MDA is a common oxidative factor indicative of lipid peroxidation. It is involved in cross‐linking of membrane compounds, thus impacting ion exchange which leads to altered enzymatic activity as well as ion permeability [40]. SOD is a resistant endogenous antioxidant enzyme, a free radical scavenger that works by converting superoxide radicals into hydrogen peroxide and water whereas CAT an antioxidant enzyme belonging to the oxidoreductase group converts hydrogen peroxide to water and oxygen [41]. In contrast, In PWE, several studies reported reduced activity of SOD and CAT while an increase in the brain concentrations of MDA which is in line with the outcomes of this study. BRV 10 + PRO 10 therapy facilitated oxidant/antioxidant balance as evidenced by the decrease in MDA levels while increase in the activity of SOD and CAT enzymes in the treated brains as collated to epileptic animals.

Modulation of the MGBA generates neuroactive signaling molecules that promote neurogenesis, and modulate the colonic and neurologic inflammation which in turn prevent cellular apoptosis. The key metabolites generated by symbiotic bacteria are SCFAs which ensure gut barrier integrity via tight junction proteins [42]. SCFAs communicate with the brain indirectly through the vagal or circulatory system prompting the release of GABA and gut hormones. Additionally, it also controls microglia activation, neuronal architecture, and neurotrophic factor levels which is implied in the attenuation of neuroinflammation [43]. Our results presented severe degenerative changes in the morphology of neuronal cells in the CA1 and CA3 regions in PTZ control. In contrast, probiotic supplementation with BRV demonstrated preserved cytoarchitecture in pyramidal cell layers of CA1 and CA3, strengthening the role of probiotics in halting excitatory drive and associated neuronal loss.

## 5 Conclusion

The current study assessed the effect of rich and long‐term probiotic administration along with BRV 10 in an animal model of generalized epilepsy and unraveled that add‐on probiotic intervention remarkably hindered epileptiform activities. Moreover, oral bacteriotherapy also mitigated PTZ‐induced psychological perturbations as well as restored oxidant/antioxidant balance. Furthermore, the BRV 10 + PRO 10 combination slowed the epileptogenic process and improved neuronal survival as few apoptotic cell bodies were seen in the pyramidal layers of CA1 and CA3. Our outcomes provide supportive evidence in favor of probiotic‐rich supplementation as an adjunctive therapy with existing ASM as these commensal bacteria might provide prophylaxis against intractable seizures, particularly in predisposed individuals.

## Author Contributions

Imran Imran and Faleh Alqahtani designed the study. Muhammad Usman Shakoor, Fashwa Khan Tareen, Zohabia Rehman, Waseem Ashraf, and Khaled Ahmed Saghir performed the experimental work. Zohabia Rehman, Waseem Ashraf, Syed Muhammad Muneeb Anjum, and Tanveer Ahmad performed data analysis. Muhammad Usman Shakoor, Fashwa Khan Tareen, Zohabia Rehman, and Waseem Ashraf prepared the manuscript draft, including the figures. All authors reviewed, edited, and approved the final manuscript.

## Ethics Statement

All experiments were carried out after getting ethical approval for animal usage from the Department of Pharmacology Ethics Committee (EC/06‐PHL‐S‐23; dated October 17, 2023) BZU, Multan. All animal experiments were conducted as per the ARRIVE guidelines.

## Conflicts of Interest

The authors declare no conflicts of interest.

## Supporting information


Appendix S1.

## Data Availability

The data that support the findings of this study are available from the corresponding author upon reasonable request.
