# Supplementary material for: Probiotics by Modulating Gut–Brain Axis Together With Brivaracetam Mitigate Seizure Progression, Behavioral Incongruities, and Prevented Neurodegeneration in Pentylenetetrazole‐Kindled Mice
Source: CNS Neurosci Ther. 2024 Oct 29;30(11):e70078. doi: 10.1111/cns.70078 (PMC11520030; doi:10.1111/cns.70078)
Supplement: Supplementary file 1 — Appendix S1. [file CNS-30-e70078-s001.docx]

**Probiotics by modulating Gut-Brain Axis together with brivaracetam mitigate seizure progression, behavioral incongruities and prevented neurodegeneration in pentylenetetrazole-kindled mice**

Muhammad Usman Shakoor^1^, Fashwa Khan Tareen^1^, Zohabia Rehman^1^, Khaled Ahmed Saghir^1^, Waseem Ashraf^1^, Syed Muhammad Muneeb Anjum^2^, Tanveer Ahmad^3^, Faleh Alqahtani^4^, Imran Imran^1*^

1. **Materials and methods for biochemical analysis**
   1. **Malondialdehyde (MDA)**

To evaluate TLE-induced lipid peroxidation, levels of MDA were analyzed calorimetrically. Briefly, 100 µl of brain supernatant was added to TBA: TCA in an equal ratio of 1:1. The reaction mixture was boiled in a water bath maintained at 100 ℃ and then cooled followed by centrifugation at 10000 g for 10 min at 4℃. Lastly, the reading in duplicates was taken at 532 nm with the microplate reader (Spectramax 340 PC384 by Molecular Devices, CA, USA) at 532 nm and MDA levels were individually normalized with protein content for all rat brains [1,2].

- 1. **Catalase (CAT)**

5% K_2_CR_2_O_7_-acetic acid solution (1:3 by volume) and 0.2 M H_2_O_2_ solution were prepared freshly. The mixture comprising tissue homogenate, phosphate buffer, and H_2_O_2_ was incubated (37 °C) for 90 s followed by the addition of dichromate acetic acid reagent, which stopped the reaction and changed the color to blue. This mixture was boiled (100 °C) for about 15 min, which changed the reaction mixture color to green. After centrifugation at 4 °C at 2500 rpm for 5 min, the absorbance was noted in the microplate reader (Spectramax 340 PC384 by Molecular Devices, CA, USA) at 570 nm. The blank and standard were simultaneously run with the test brain homogenates, and the catalase activity was measured using the 43.6 M^−1^ cm^−1^ as the extinction coefficient for H_2_O_2_. Outcomes were expressed as µmol/min/mg of protein [3].

- 1. **Superoxide dismutase (SOD)**

To assess SOD activity, 50 µl of 50 mm Na_2_CO_3_ (Sodium carbonate, Sigma, Aldrich), 20 µl of 0.1 mm EDTA (ethylene diamine tetraacetic acid, Sigma, Aldrich), and 40 µl of 0.56 mM of NBT (nitro blue tetrazolium, Molekula, England) was added to 50 µl of brain supernatant. Then 40 µl of 0.1 mM HAC (hydroxyl amine chloride) was added to the reaction mixture and sample readings in duplicate were observed for up to 45 minutes with an interim of 5 min [4,5].

**REFERENCES**

1. Chow, C.K.; Tappel, A.L. An enzymatic protective mechanism against lipid peroxidation damage to lungs of ozone-exposed rats. *Lipids* **1972**, *7*, 518–524.

2. Haider, S.; Naqvi, F.; Batool, Z.; Tabassum, S.; Sadir, S.; Liaquat, L.; Naqvi, F.; Zuberi, N.A.; Shakeel, H.; Perveen, T. pretreatment with curcumin attenuates anxiety while strengthens memory performance after one short stress experience in male rats. *Brain Res. Bull.* **2015**, *115*, 1–8.

3. Farjad, E.; Momeni, H.R. Silymarin ameliorates oxidative stress and enhances antioxidant defense system capacity in cadmium-treated mice. *Cell J.* **2018**, *20*, 422–426.

4. Chidambara Murthy, K.N.; Jayaprakasha, G.K.; Singh, R.P. Studies on antioxidant activity of pomegranate (punica granatum) peel extract using in vivo models. *J. Agric. Food Chem.* **2002**, *50*, 4791–4795.

5. Naskar, S.; Islam, A.; Mazumder, U.K.; Saha, P.; Haldar, P.K.; Gupta, M. *In vitro* and *in vivo* antioxidant potential of hydromethanolic extract of *phoenix dactylifera* fruits. *J. Sci. Res.* **2010**, *2*, 144–157.
